# A qualitative study examining newly diagnosed breast cancer patients’ experiences of participating in the Alberta Moving Beyond Breast Cancer (AMBER) prospective cohort study

**DOI:** 10.1186/s12885-023-10967-3

**Published:** 2023-06-02

**Authors:** Lynn Corcoran, Christine M. Friedenreich, Margaret L. McNeely, Nicole S. Culos-Reed, Gordon Bell, Leanne Dickau, Kerry S. Courneya, Jeff K. Vallance

**Affiliations:** 1 Faculty of Health Disciplines, Athabasca University, 1 University Drive, Athabasca, AB T9S-3A3 Canada; 2 Department of Cancer Epidemiology and Prevention Research, Cancer Care Alberta, Alberta Health Services, Calgary, AB Canada; 3 Department of Oncology, Cumming School of Medicine, University of Calgary, Calgary, AB Canada; 4 Department of Community Health Sciences, Cumming School of Medicine, University of Calgary, Calgary, AB Canada; 5 Department of Physical Therapy, University of Alberta, Edmonton, AB Canada; 6 Faculty of Kinesiology, University of Calgary, Calgary, AB Canada; 7 Faculty of Kinesiology, Sport, and Recreation, College of Health Sciences, University of Alberta, Edmonton, AB Canada

**Keywords:** Breast cancer, Prospective cohort, Qualitative, Recruitment, Physical activity

## Abstract

**Background:**

Decisions to participate in cancer trials are associated with uncertainty, distress, wanting to help find a cure, the hope for benefit, and altruism. There is a gap in the literature regarding research examining participation in prospective cohort studies. The aim of this study was to examine the experiences of newly diagnosed women with breast cancer participating in the AMBER Study to identify potential strategies to support patients’ recruitment, retention, and motivation.

**Methods:**

Newly diagnosed breast cancer patients were recruited from the Alberta Moving Beyond Breast Cancer (AMBER) cohort study. Data were collected using semi-structured conversational interviews with 21 participants from February to May 2020. Transcripts were imported into NVivo software for management, organization, and coding. Inductive content analysis was undertaken.

**Results:**

Five main concepts associated with recruitment, retention, and motivation to participate were identified. These main concepts included: (1) personal interest in exercise and nutrition; (2) investment in individual results; (3) personal and professional interest in research; (4) burden of assessments; (5) importance of research staff.

**Conclusions:**

Breast cancer survivors participating in this prospective cohort study had numerous reasons for participating and these reasons could be considered in future studies to enhance participant recruitment and retention. Improving recruitment and retention in prospective cancer cohort studies could result in more valid and generalizable study findings that could improve the care of cancer survivors.

## Introduction

In Canada, 28,600 women were expected to be diagnosed with breast cancer in 2022, and over 5,000 women are expected to die from the disease [1]. Having those affected by breast cancer participate in cancer research is important to maximize the validity and reliability of research findings. Previous research related to recruitment, retention, and motivation to participate in cancer research has mainly focused on clinical trials [2–7]. Less is known about these factors in the context of prospective cohort studies. Compared with clinical trials, prospective cohort designs offer advantages, including the ability to examine multiple exposures over time, as well as longer-term endpoints including recurrence and death. Unlike intervention studies, there is no immediate benefit to participation in a prospective cohort study (e.g., participants are not receiving an intervention) which may impact participant retention in the study. Consideration of recruitment, retention, and motivation to participate in this type of study design may facilitate a better understanding of why women recently diagnosed with breast cancer might participate and engage in a longer-term cohort study. The integrity and robustness of large-scale prospective cohort studies depend on the retention of participants at all data collection time points. Exploring participants’ experiences in these studies may help researchers determine which factors influence an individual’s consent to participate and their ongoing commitment to the study over potentially several years.

Recruiting and retaining diverse groups such as older adults, women, racially and ethnically diverse groups, and/or patients with specific types of cancer, has also been studied [8, 9]. These studies reported a decrease in the recruitment of minorities, older patients, and cancers including melanoma, lung, and pancreatic, over time. Recent reviews, meta-analyses [4, 5], and meta-syntheses [3, 6] have examined the growing body of literature related to participation in cancer clinical trials. These studies found decisions to participate in cancer trials are associated with uncertainty, distress, wanting to help find a cure, the hope for benefit, and altruism. There is a gap in the literature regarding research examining participation in prospective cohort studies.

We are currently conducting the Alberta Moving Beyond Breast Cancer (AMBER) Study, a dual-site prospective cohort study designed to measure the role of physical activity, sedentary behaviour, and health-related fitness on breast cancer outcomes [10, 11]. Women newly diagnosed with stage I (*≥* T1c) to stage IIIc breast cancer (N = 1,528) were approached after surgery (and before the start of adjuvant therapy) to participate in the AMBER Study. After consenting to the AMBER Study, participants underwent comprehensive assessments including the completion of detailed health and lifestyle questionnaires (regarding lifestyle, physical activity, and diet), one to two days of in-person fitness testing including muscular strength and endurance tests as well as aerobic fitness, a blood sample, screening for lymphedema, a body composition measurement using dual x-ray absorptiometry scan, and monitoring physical activity and sedentary behaviour for one week using two accelerometers. Data are collected at three time points: baseline (one to two months following breast surgery) and at intervals of one and three years. At the five-year follow-up time point, only the questionnaires are completed.

The aim of this study was to examine the experiences of newly diagnosed women with breast cancer participating in the AMBER Study to identify potential strategies to support patients’ recruitment, retention, and motivation. The insights gained from this study may inform investigators engaged in other observational prospective cohort studies.

## Methods

### Study Design

A qualitative approach using an inductive content analysis design was undertaken in this study. This approach is well-suited for eliciting meaning and understanding peoples’ lived experiences [12, 13]. Inductive content analysis involves identifying codes within the data itself, adding to, and adapting the list of codes throughout the analytic process. This process is used when little is known about the topic being explored or when the phenomenon is complex and not well-defined [12, 14]. The process of inductive content analysis results in several main concepts (content categories) with sub-concepts (subcategories) emerging as extensions from each of the main concepts.

### Recruitment, sample, and sampling

The AMBER Study newsletter (https://www.amberstudy.com/newsletters.html) was used to advertise and recruit participants for this study. Recruitment began in January 2020 and concluded in May 2020. The AMBER newsletter is an online newsletter that is sent out to all participants quarterly. The newsletter contains updates on the AMBER Study, AMBER participant profiles which highlight an individual participant’s perspective often including their results, progress, or goals, messages from the study investigators, and general news about breast cancer. Maximum variation sampling, a type of purposive sampling was implemented as the sampling strategy because it helps to identify important patterns across cases in the context of heterogenity of participants [15]. Maximum variation purposive sampling was done based on study location (i.e., Edmonton or Calgary) and participants’ age, length of time in the cohort study, and cancer stage at diagnosis. An information letter and consent form were emailed to potential participants and completed before the interview. Participants in the AMBER study did not receive material incentives that research participants may commonly receive (e.g., money, t-shirts, gift cards).

### Data collection

Data were collected using semi-structured conversational interviews via telephone (LC) with 21 participants from February to May 2020. Robust and diverse opinions and feedback from participants were obtained, and the researchers determined that data saturation was achieved with the sample’s 21 participants. Researchers based this determination on the following principles: analysis of patterns in the data during collection and analysis to the point whereby no new data/concepts emerged [15, 16]; use of purposive sampling to recruit participants with the knowledge and experience to provide a rich description in addressing the research question [15, 17]; and sufficient detail regarding the method to potentially replicate the study [16, 17]. The semi-structured interviews were designed to understand the topic from the participant’s perspective and to explore the meaning of their experiences [18]. Interviews were up to 45 min in length, followed an interview guide (Table 1), and were digitally recorded. The interviewer further documented the interviews and initial impressions of the interview content through field notes. All participants were asked if they were willing to participate in a follow-up phone call scheduled one week after their initial interview to garner any additional insights. A follow-up call was completed with 20 of 21 participants but few additional insights were shared. A research assistant transcribed the interviews verbatim.

**Table 1:** Guide for semi-structured interviews in the AMBER cohort study

1.	A diagnosis of breast cancer is a significant life event. Tell me about what this diagnosis was like for you.
2.	Tell me about how you became involved with the AMBER Study.
3.	How did you decide to participate in the study?
	**Probes**: What influenced your decision to participate?
	What concerns did you have regarding participating in the study?
4.	As a participant in the AMBER Study, tell me about the recruitment process.
	**Probes**: Did you receive any recruitment materials/a letter?
	Did you receive any follow-up such as a phone call?
5.	Have you ever felt like quitting the AMBER Study?
	**Probes**: What was happening that made you consider this action?
	How did you decide to continue with the study?
	What influenced this decision?
6.	Is there anything else you would like to add, related to what we’ve been discussing so far?
7.	Are there any questions I haven’t asked that you think would be important for me to ask?
8.	Do you have any questions?

### Data Analysis

Transcripts were imported into NVivo 12 (QSR International, Doncaster, UK) software for management, organization, and coding. The inductive content analysis process including the three phases of data reduction, data grouping, and formation of concepts was undertaken [14]. Independently, two researchers read and re-read the transcripts (LC and JV). Open codes were initially identified from the raw data. Researchers worked separately with the data and then discussed and developed main concepts and sub-concepts. Field notes were reviewed by one researcher (LC) throughout the data analysis phase to determine convergence or divergence with emerging concepts.

### Ethical considerations

#### Ethics approval

was obtained through the Health Research Ethics Board of Alberta: Cancer Committee (HREBA.CC-17-0576). Participants’ anonymity was ensured by assigning them a number and removing identifiers from transcripts. Data were stored on a password-protected and encrypted computer. Digital recordings were deleted following the transcription of interviews.

## Results

The demographic, clinical, and health behaviour characteristics of the study sample were similar to the full cohort of participants [11] (see Table 2). The mean age of this subset was 55.8 years (SD = 13.8), 61% were from Edmonton, most were married or common-law (n = 19), Caucasian (n = 20), had an undergraduate or graduate school education (n = 13), and worked less than 35 h per week (n = 14). The mean body mass index (BMI) of the sample was 28 kg/m^2^ and they had an average of 70 min of at least moderate intensity total activity per day (which included household, recreational, occupational, and transport). Most women were diagnosed with stage II or III breast cancer (n = 16) and 14 women received chemotherapy. The demographic, clinical, and health behaviour characteristics of the study sample were similar to the other 1,507 AMBER participants [11] (see Table 2). In our sample, there were significantly fewer participants with stage I disease and more participants with stage III disease compared to the remaining cohort. Also, a significantly greater percentage of participants in this study received neoadjuvant therapy compared to the remaining cohort.

**Table 2:** Demographic, clinical, and health behaviour characteristics of AMBER qualitative study participants (n = 21) compared to the AMBER cohort study participants (N = 1,507)

Characteristic	Qualitative sample		AMBER cohort		P
	(N = 21)		(N = 1,507)		
	N (%)	Mean ± SD	N (%)	Mean ± SD	
**Demographic**					
Age at diagnosis		55.8 ± 13.8		55.4 ± 10.8	0.876
Study location					
Edmonton	13 (61.6)		632 (8.9)		0.08
Calgary	8 (38.1)		875 (12.1		
Marital Status					
Married or common-law	19 (90.5)		1113 (72.8)		0.206
Widowed/separated/divorced	1 (4.8)		284 (18.6)		
Single/never married	1 (4.8)		110 (7.2)		
Ethnicity					
Caucasian	20 (95.2)		1311 (85.8)		0.364
First Nations/Indigenous/Metis	1 (4.8)		12 (1)		
Education					
High school or below	2 (9.5)		3412 (22.4)		0.142
College	6 (28.6)		481 (31.5)		
University	5 (23.8)		393 (25.7)		
Graduate school	8 (38.1)		291 (19)		
Annual Family Income					
<$50,000	2 (9.5)		245 (16)		0.570
50-100k	5 (23.8)		486 (31.8)		
100-150k	6 (28.6)		353 (23.1)		
>150k	8 (38.1)		423 (27.7)		
Employment Status					
Works < 35 hours per week	14 (66.7)		1006 (65.8)		0.993
Works *≥* 35 hours per week	7 (33.3)		501 (32.8)		
**Clinical/behavioural**					
Body mass index (kg/m^2^)		28 ± 4.8		27.5 ± 5.6	0.716
Normal weight	6 (28.6)		572 (37.4		0.378
Overweight or obese (BMI > 25 kg/m^2^)	15 (71.4)		935 (61.2)		
Waist circumference (cm)		95.6 ± 14.8		92.8 ± 13.5	0.351
Waist-to-hip ratio (cm)		0.89 ± 0.08		0.88 ± 0.07	0.291
% body fat		42 ± 6.7		43.1 ± 7.2	0.481
Resting heart rate		68.2 ± 9.9		72.4 ± 10.1	0.06
VO_2_max (ml/kg/min)		26.2 ± 6.1		25.9 ± 6.2	0.852
MVPA^†^ minutes per day		69.6 ± 50.3		61.1 ± 33.8	0.263
Number of 1st degree relative breast cancer family history		0.4 ± 0.7		0.3 ± 0.6	0.983
Stage					
I	5 (23.8)		677 (44.3)		**< .001**
II	9 (42.9)		703 (46)		
III	7 (33.3)		127 (8.3)		
Histology					
Ductal carcinoma	15 (71.4)		1275 (83.4)		0.272
Invasive ductal and lobular					
carcinoma mixed	2 (9.5)		56 (3.7)		
Invasive lobular carcinoma	4 (19)		159 (10.4)		
Mastectomy					
Yes	10 (47.6)		622 (40.7)		0.558
No	11 (52.4)		885 (57.9)		
Chemotherapy					
Yes	14 (66.7)		877 (57.4)		0.434
No	7 (33.3)		630 (41.2)		
Neoadjuvant therapy					
Yes	4 (19)		113 (7.4)		**0.048**
No	17 (81)		1394 (91.2)		
Comorbidity score (0–8)		1.0 ± 1.4		.92 ± 1.1	0.733
Smoking					
Never smoker	13 (61.9)		855 (56)		0.643
Past smoker	8 (38.1)		541 (35.4)		

Data are presented as the mean (standard deviation) for continuous variables and frequency (percentage) for categorical variables. SD, standard deviation.

Groups were compared using chi square for categorical data and analysis of variance for continuous data.

^†^ Moderate and vigorous physical activity minutes as assessed by accelerometry.

Five main concepts associated with recruitment, retention, and motivation to participate in the AMBER Study were identified during the interviews. These included: (1) personal interest in exercise and nutrition; (2) investment in individual results; (3) personal and professional interest in research; (4) burden of assessments; (5) importance of research staff.

### Personal interest in exercise and nutrition

Many participants spoke of being interested in their overall health and in particular, their exercise or nutrition. Often, participants were interested in both and that motivated them to participate in AMBER: *“I joined the AMBER Study because I wanted to find out more about myself and what I can do to improve myself, too.* [I wanted to] *change my health, the direction my health is going” (P5).* Sub-concepts in this category were beliefs regarding the importance of exercise and awareness of the importance of nutrition.

#### Beliefs regarding the importance of exercise

Most participants reported being interested and engaged in a range of physical activities including walking, biking, and swimming as well as outdoor pursuits such as skiing and hiking. When asked about her reasons for participating in the AMBER Study, P2, who had completed five years of data collection stated, *“…my own personal and professional belief is that physical activity is so very important to overall health and well-being.”* For P17, physical activity was seen as part of an entire picture of treatment and recovery from breast cancer.

*“It seemed like a really natural thing for me. You want to do treatment. You want things to be better long term and better for others. When I heard there was exercise involved and testing, then I thought, ‘Well that can only be a good thing.’” (P17)*

When the COVID-19 pandemic resulted in restrictions on indoor fitness, P20 demonstrated her commitment to physical activity:

*“We live out in the country. My sisters come out and we turn up the music and we dance. We have been dancing out on the lawn. Not pretty* [chuckle] *but, we get exercise.” (P20)*

While an interest in remaining physically active was frequently reported as a reason for participating in the AMBER Study, the awareness of the role of nutrition in overall health was also articulated.

#### Awareness of the importance of nutrition

Most participants had a keen awareness regarding the importance of nutrition and its influence on their overall health. In particular, they made reference to the detailed nutrition questionnaire used in the study (i.e., Canadian adaptation of the US National Cancer Institute’s past year Diet History Questionnaire II, CDHQ-II) [17] that was administered four times (at baseline and years 1, 3 and 5). Simply completing the questionnaire seemed to have an influence on P16, indicating that she was more mindful of her nutrition choices.

*“It made me stop and think when I did the questionnaire. It made me stop and think about what I ate and maybe what I shouldn’t have eaten* [chuckle]. *I laugh when I say that because I mean, I was so good with all my vegetables, and I cut back on a lot of stuff. Then all of the sudden it’s like, ‘I’m going to have a little bag of chips’* [chuckle] *and it’s like, ‘Wait a second,’ and you think more and make better or wiser choices because you really look at everything you ate, like right down to the condiments you put on your sandwiches.” (P16)*.

The CDHQ-II is a detailed food frequency questionnaire consisting of 134 food items and eight dietary supplement questions that takes approximately one hour to complete [19]. While participant feedback related to the length of this questionnaire is addressed below, the impact of simply completing this questionnaire on eating habits was apparent as stated by P10 and P5:

*“This study changed my eating habits. It changed my thinking and my life. Even the questions on the questionnaire sort of tweak you like, ‘Do you drink V8 juice or this juice or that juice?’ The questions themselves were good, because a lot of times you don’t even think about what kind of juices you’re drinking.” (P10)*

*“You know what? I really enjoyed doing the questionnaires because it really made me conscious of the choices I’m making as far as exercise and activity and eating. It’s like a real good reminder.” (P5)*

### Investment in individual results

As the participants expressed their beliefs and interest in exercise and nutrition as motivation for taking part in the AMBER Study, they were also interested in their individual results. P3 stated, *“I really quite enjoyed it, all the physical strength testing. I enjoyed it quite a bit. I learned a lot about me.”* P18 agreed with this statement adding: *“You know for me personally it was fascinating to see in those periods between the testing how much better or worse I was at something.”* Sub-concepts in this category were data as impetus for change and data as personal motivation. The participants’ individual results were shared with them in written documents along with an explanation from the AMBER Study Clinical Exercise Physiologist/Research Coordinator at baseline, 1-, and 3-years.

#### Data as impetus for change

When a study is conducted over the course of five years following a diagnosis of breast cancer, there is an opportunity to examine and change certain aspects of lifestyle habits. Participants were interested in their results, with one participant stating that her motivation was *“…partly selfish, I guess. I thought it would be a good way to see how I change, you know, from the very beginning to five years later” (P11).* Another participant alluded to the ongoing nature of the testing and the results.

*“I really enjoyed learning about myself. The report on how fit you are and how obese you are, and how well you did on the physical testing. That was very, very interesting and very motivating for me, like, I can’t wait for next time to find out where I am. What did I do? Did I gain weight? Did I lose weight? How much can I do now? How much can I lift now? All that stuff was very satisfying to me.” (P3)*

Having completed three rounds of data collection, P12 was interesting in comparing her data over time and in the context of what was happening in the trajectory of her breast cancer process:

*“I found it was worth it and I actually really like the information that it provided me with, and I like being able to compare the information from the three different years. The one that I did last year was right after all my reconstructive surgery.* [I] *found out my* [results from] *the physical testing*, [I] *got my own personal information.” (P12)*

#### Data as personal motivation

With the set structure of periodic time points related to data collection, these time points served to motivate the participants. Knowing that they would be tested and ultimately accountable for their results functioned as inspiration to think about and follow through with health behaviours in the areas of nutritious eating and regular physical activity. P16 found that the study motivated her to think more about healthy nutrition.

*“Participation in the study got me motivated to be more aware of exercising and eating properly like vegetables are more important than that chocolate bar that I used to love* [chuckle]. *I think the AMBER Study keeps you conscious of what you eat because I went* [to the study] *after surgery, just about a month or two later, and then within a year’s time. It brings up everything again, so you kinda touch base again. ‘Are you aware of this? Or are you looking at this?’” (P16)*

P3 and P9 spoke about the relationships between testing, getting results, and their motivation to progress and improve related to exercise and overall health.

*“I just thought this would motivate me to keep going and you know, trying to keep my results up as much as I can. It gave you a time frame. I think it encourages me knowing in the back of my mind that I will be tested - it encourages me to keep going - keep walking. I lifted weights as soon as I could after the surgery. I borrowed my daughter’s 5 pound weights to lift, so that I wouldn’t get lymphedema.” (P3)*

*“Just to see your own progress over time. So, I participated in June, and I had X range of motion. Come this June, I participate again so, ‘Have I increased my range of motion? Have I increased my cardiovascular ability? Have I improved myself?’” (P9)*

### Personal and professional interest in research

The participants often spoke about the importance of contributing to research. Some participants had an understanding and appreciation for research as well as a shared belief in supporting research. Contributing to research was interwoven with altruism and helping other people. *“It’s all about the research and helping, helping get it done, helping other people” (P3).* The potential of advancing the body of knowledge to benefit others was evident: *“I wanted to be able to help with maybe new discoveries and anything that will down the road might help other women” (P18)* and *“I’m interested in supporting the research. When the research opportunities came into the room, literally… I just said yes to everything” (P2).* Sub-concepts in this category were supporting and believing in research as well as altruism. These sub-concepts are presented separately to reflect emphasis related to patterns in the data however, it can be seen in these data that supporting research was inextricably connected with altruism.

#### Supporting and believing in research

Many participants spoke of wanting to support research as well as believing in the value of research in terms of contributing to science.

*“I thought it* [this study] *was important. I felt the work that they do was important. If I can contribute, I was excited to do it. What I’m trying to say is that I believe in participating to help with science, I believe in those steps and so any way I can help is important to me. (P5)*

Interestingly, and perhaps reflective of the level of formal education of participants, several had completed graduate studies, such as P13 and P14, which gave them an even deeper appreciation of research:

*“I just finished my Master’s, so I have a lot of respect for research. I wanted to support that, and I wanted to support anything that could help somebody in my shoes, that was going through what I was going through.” (P13)*

*“Any time I can participate in anything at all, it helps the researchers, it helps the cause, breast cancer. There was no question in my mind. Research is research, just like yours. I thought if I could be of some help, I will be of some help. I did a Master’s thesis and I needed subjects and I was so happy to have subjects and so* [now] *I was willing to be a subject.” (P14)*

#### Altruism

As noted in the subtext of the quotes in the previous section, most participants expressed a concern for the welfare of others, in particular, other women with breast cancer. Most participants spoke of altruism, using words such as *help*, *helping*, and *giving back*. Notably, there was reference to participating in the study as part of a historical and collective effort by referring to *women* and *ladies* who had participated in research studies *before me*. These words and phrases reflected an appreciation for past research and the participants who contributed to those studies.

*“I wanted to be in a study because I felt that many women had gone before me and done a lot of studies and I wanted to be part of helping people in the future. That was of paramount importance. Look, you know, you got this terrible diagnosis, but you know it’s time to give back and you think that you are going to give back and you will, but you will gain more, much, much more than you thought you would. (P10)*

There was some reference to the AMBER Study in particular. As a prospective cohort study, there was no treatment or intervention perhaps giving participants the impression that involvement in this study was not arduous.

*“I just thought I could be part of the research into breast cancer and not necessarily the disease itself, but you know, how to recover and things like that. It seemed like a pretty good thing to do and* [it is] *not invasive, it didn’t involve a lot of stuff. They took blood at the beginning of the AMBER Study. It just seemed like a valuable thing to do. (P7)*

### Burden of assessments

In answering the questions and prompts regarding any concerns about participating in the AMBER Study, many participants stated that they had no concerns or worries related to taking part in the study. However, it remains important to explore the challenging aspects of participating in a five-year study. Three participants at the time of data collection, had *partial* data collected at 1-, 3- and 5-year data points, respectively. Two participants were coded as *refused* in terms of their data collection at the 3- and 5-year time points, respectively. Sub-concepts in this category were challenges related to completing the lengthy nutrition questionnaire and issues related to the maximal cardiopulmonary fitness test.

#### Lengthy nutrition questionnaires

Most participants who expressed any concerns about participating in the AMBER Study mentioned challenges related to completing the lengthy nutrition questionnaire, the CDHQ-II. *“I was sitting there for hours answering those questions* [chuckle]. *I was sitting for hours on the couch, answering questions about what the hell I eat and trying to remember, what I eat for the last year was horrible” (P3).* The paper-based version of the CDHQ-II is 40 pages in length [19, 20]. Additionally, participants found the questions challenging either in terms of their specific diet or in terms of picking the best fixed-choice response.

*“I was going through the diet* [questionnaire]. *I’m a vegetarian. I’ve been a vegetarian for years and years. Then there are pages and pages of like, ‘Do you eat ham? Do you eat processed lunch meat? Do you eat steak? Do you eat roast beef? How many times a week?’ I was just like, could there just be a question and like, ‘skip this section’ instead of no, no, no, no, no, no, when you have to fill it in with the little bubbles and you fill it in with your pencil no, no, no, no a million times no because I don’t eat any meat.* [laughter] *It gets a little frustrating when you have to answer that.” (P13)*

*“And then of course there is the* [diet] *questionnaire. I found some of the questions are a little bit ambiguous in that there was a lot you could answer this way and you could have answered them another way and each way would probably give, whoever was compiling the data, a different point of view.* [I thought], *‘Hmm, okay, now what is the best way for me to answer this?’” (P18)*

Memory issues as a result of chemotherapy also added to the challenge of completing the diet questionnaire:

*“That* [diet] *questionnaire is so long, by the time you get a quarter of the way through it, you’re starting to feel confused, or I was. I would just have to put it away. Go away from it and I know that made it harder in some respects to start over again. I found that there was a lot of paperwork. It was very difficult because of chemo brain among other things. They would ask things about, you know, over the last year*, [there were] *food related questions and those were hard. My memory is still not recovered so trying to remember over the course of a year was not easy.” (P17)*

#### Cardiopulmonary fitness test

This maximal fitness test involved participants walking on a treadmill and wearing a gas exchange mask. Understandably, this test presented challenges for participants including issues related to not being familiar with this test and wearing a mask.

*“I was a little intimidated by the treadmill ‘cause they put that air mask on and then you learn to breathe* [chuckle]. *You walk first and then they keep speeding it up and then they put on a bigger incline and then they just say, ‘Raise your hand when you’ve done enough.’ Because I’d never* [done this], *it’d be like* [asking] *you to scuba dive, you know? (P20)*

*“I think, the most difficult thing for me that first time around was the VO*_*2*_*max test.* [I] *had a weird kind of mask on me at that time. They used a different one the second time. But the first one, was really bothering me and the smell of the sanitizer was really strong, so I was inhaling all these fumes and so that was really the only complaint I have.” (P13)*

While the mask was also an issue for P21, the notion of doing the maximal cardiopulmonary fitness test “to failure” was ambiguous:

*“I found the treadmill to be quite… I think I probably gave up before they wanted me to give up* [chuckle]. *I found that mask so awful, I really did. I thought I’d probably could have done better… I also found it started out really slowly…it wasn’t something I really liked. (P21)*

After becoming familiar with what was involved in cardiopulmonary fitness test and then experiencing doing this test, P15 found that she had to convince herself into doing this test on subsequent data collections following baseline, *“I had to mentally talk myself into doing the VO*_*2*_*max.” (P15)*.

### Research staff matter

The interactions with the research study staff mattered to participants. How staff conducted themselves when obtaining consent, collecting data, and sharing data was important. The women in the AMBER cohort study are a vulnerable population having received a diagnosis of breast cancer and undergone treatment including surgery and possibly radiation and chemotherapy. As such, communication including what staff said and how they said it to participants was critical. The comportment of the AMBER Research Study Coordinators as well as research study staff was mentioned by several participants:

“[The research study coordinator] *was the administrator of the project and she and the people who worked for her were excellent.” (P21)*

*“It was so nice to interact with* [the research study coordinator] *and one of the other testers. It was just really very embracing; I’m going to say. You had a chance to talk to someone that understood.” (P2)*

While obtaining informed consent may seem routine for those involved in research, P19 appreciated the way this step was presented to her, “*I remember that she* [the research study coordinator] *was very thoughtful, the way she contacted me. She said, ‘You have the right to refuse anytime.’ It* [participation in the study] *was all on my terms” (P19).* P10 acknowledged the profound impact of what the study coordinator said to her:

*“When I met* [the research study coordinator] *the first words that she said changed my life…I just understood, it was like a key being turned in a lock. It’s* [breast cancer] *not a death sentence. You can survive this, and you can thrive and do really well. So, she taught me in a nanosecond from her response to me and that was more important than all of everything else that proceeded from the AMBER Study.” (P10)*

## Discussion

The results of this study suggest that participants in a longer-term prospective cohort study have a variety of reasons for participating and these reasons can inform recruitment and retention. The first three emergent themes (i.e., personal interest in exercise and nutrition, investment in individual results, and personal and professional interest in research) were closely related to study recruitment whereas the last two themes (i.e., burden of assessments, and importance of research staff) were most closely related to retention.

The participants in this study were interested in a variety of health behaviours including physical activity and nutrition. Participants were involved in structured and unstructured exercises occurring both indoors and outdoors. Participants’ belief in the importance of physical activity and their commitment to exercise made the topic of this study appealing. Taking an interest in one’s personal health is a motivating factor for participation in observational research [21] and people with a history of cancer are more likely to participate when the study is personally meaningful [22]. While physical activity following a diagnosis of breast cancer is related to improved breast cancer survivorship [23, 24], participants did not report a history of cancer as influencing their decision to join the AMBER Study. Even though a healthy diet is associated with improved outcomes for women with breast cancer [25] participants did not mention this association as a reason for their participation. The act of completing the food frequency questionnaires led participants to consider their dietary choices carefully with some indicating that completing this questionnaire changed their dietary intake habits. One of the reasons participants consent to a study is because they find the topic to be of interest and in alignment with both their personal values and lifestyle.

Participants were interested in their individual results related to parameters of health and fitness. Individualized data including body composition, functional capacity, bone density, and musculoskeletal/aerobic capacity collected and shared with the AMBER Study participants served as an impetus for making changes and providing motivation for participants. The use of incentives is a common strategy to recruit and retain research study participants [26, 27]. Participants in the AMBER study did not receive material incentives that research participants may commonly receive (e.g., money, t-shirts, gift cards). However, our findings suggest that receiving free health and fitness tests (that one cannot easily access) was perceived as a significant incentive to participate in the AMBER study. Reconceptualizing personalized health data as an incentive is worthy of consideration for researchers planning and designing longer-term studies. Despite not receiving an intervention in observational studies, the process of measuring and sharing this information with participants may indeed function as an intervention as they may act and make changes based on their results.

It is important to be mindful of how results are shared with participants. All study participants’ results were shared with them after testing at baseline, 1- and 3- years. The Clinical Exercise Physiologist reviewed and interpreted the results of the two days of testing. Participation in research is more likely when participants receive personalized information relevant to themselves [28]. Quarterly updates on the AMBER Study are shared via an online newsletter emailed to all participants. In addition to the cohort study updates, a section in the newsletter entitled “AMBER Participant Profiles” highlights an individual participant’s perspective often including their results, progress, or goals. Our qualitative data suggest participants appreciated and valued these different types of feedback and study progress updates.

Participants spoke to the larger context of wanting to support research (in general) and breast cancer research (in particular). Some participants in this study worked in health care settings or research environments and some had completed graduate degrees. This finding is consistent with published studies examining participation where people with higher levels of formal education tend to be more willing to participate in research [29]. Participants’ knowledge of the research process and the importance of the application of research findings to clinical practice was evident and this may have motivated them to sign up and participate in the AMBER Study.

In supporting breast cancer research, participants indicated that they wanted to “help” and “give back” to other women diagnosed with breast cancer. This assertion aligns with the concept of altruism and is a motivating factor for research study participation [30–32]. Participants in the AMBER Study indicated they were helping women with breast cancer by contributing to this research. It is compelling to also consider altruism in the context of participants being interested in their individual results. Altruism is not always a discrete motivator; it is often operating in combination with personal benefit and self-interest [30, 31]. Participation in a study is more likely when people with a history of cancer perceive the study to be personally meaningful [22] and when there is a perceived personal benefit to participation [33].

Participants stated that the food frequency questionnaires were lengthy and challenging to complete. Supporting participants can consist of informing them of the length of time to set aside for participating in the study so they can plan their time and energy accordingly. Research team members could also mitigate this burden by acknowledging the effects of chemotherapy on cognitive function and fatigue [34, 35]. Suggesting strategies such as completing the questionnaire in several shorter sittings as opposed to one lengthy session may be of benefit. The maximal cardiopulmonary fitness test was also difficult and caused anxiety for some participants. The challenges of a maximal cardiopulmonary fitness test are well-recognized and have been reported in the literature in special populations including cancer populations. These challenges include pain, discomfort, anxiety, and fatigue [36, 37] experienced by the participants. The AMBER Study offered information about the tests and participants also received extensive personalized support from research staff and testers which has been determined to be important for recruitment and retention [6].

Participants spoke highly of the comportment and expertise of the AMBER Study research staff who interacted with them. Women with breast cancer are a vulnerable population and the importance of research staff communicating in a and sensitive manner is key. Trust in the physician, nurse, or research staff is instrumental to recruitment [38]. Care with doing testing such as weighing and measuring and communicating the results of these tests sensitively with women who have co-morbidities and/or pre-existing health concerns (e.g., diabetes, obesity, hypertension, eating disorders, body dysmorphia) must be prioritized. Specialized training in clear communication has been suggested as important for research staff such as physicians and nurses [6]. It is important to balance the objectivity required to adhere to research protocols while considering the personal context of each participant and the knowledge that cancer patients/survivors are a vulnerable group.

### Strengths and Limitations

Strengths of our study include the use of maximum variation sampling to recruit a sample with some diversity in terms of sociodemographic (e.g., age, rural/urban place of residence) and clinical characteristics (e.g., breast cancer stage). Another strength includes employing inductive content analysis from two independent researchers over the period of several months with periodic meetings to determine and accurately name concepts and sub-concepts. This study also has limitations that warrant mention. First, there was some selection bias since all participants were from one province and they were all Caucasian, primarily formally educated, mainly married/common-law and only two had not completed some of the assessments. Hence, the generalizability of the findings is restricted to similar populations. Second, data collection for this study coincided with the onset of the COVID-19 pandemic which might have influenced participants’ responses to the interview questions as people in general may have been experiencing feelings of uncertainty and anxiety. Future research should attempt to interview participants who drop out or withdraw from cancer studies, although there may be ethical challenges to doing so (e.g., contacting participants after they have withdrawn from the study).

## Conclusion

Our results suggest multiple factors may influence the recruitment and retention of cancer patients in a prospective cohort research study such as the AMBER Study. Our findings can be used to develop research environments that optimize participant recruitment and retention. More research on this topic has the potential to expand our understanding of the layered and complex motivations of participants in observational prospective cohort studies. In so doing, loss to follow-up in longer-term prospective cohorts with cancer patient/survivor groups and missing data may be minimized.

## Data Availability

The datasets used and/or analyzed during the current study will be made available upon reasonable request from the corresponding author; the data are not publicly available due to privacy or ethical restrictions.
